# Diseases of the Blood and Blood-Vessels

**Published:** 1905-01-28

**Authors:** 


### PROGRESS IN MEDICINE AND SURGERY.

### DISEASES OF THE BLOOD AND BLOOD-VESSELS.

(*Continued from page* 301.)

**Arterio sclerosis.**—The nutrition of the wall of arteries is maintained in one of the following ways. Either all three coats are nourished by blood derived from the vasa vasorum, or the supply of nutrition is twofold, the intima being nourished by the blood within the vessel and the remaining coats from the vasa vasorum. But, even though blood-vessels do not reach the intima, nourishment may reach it by way of lymphatics which penetrate the elastic lamina. Pathological changes in the intima may, according to Jores,[2] take the form of mere hyperplastic thickenings, in which the individual cellular elements of the inner coat proliferate and the lamellæ of the elastic layer multiply, or this may be confined to the subendothelial tissue. Jores is of opinion that these changes are due merely to exaggeration of a reaction in response to a call for increased power of resistance against enhanced blood pressure. If the demand is excessive arterio-sclerosis results, and, if still further developed, fatty degeneration takes place in the tissue which has been thrown up as a defence. Stengel[3] has found that, though the systolic blood-pressure in arteries is not necessarily high, the diastolic blood-pressure is always high until that time when the myocardium becomes diseased and fails. In examining the blood of these people one always unexpectedly finds a high blood count. One of the first evidences of arterial-sclerosis is a sustained and prolonged character of the first sound before myocardial degeneration sets in.

**Tissue-Lymph Circulation.**—In the Oliver-Sharpey lectures Dr. Oliver[4] presented the results of investigations on the tissue-lymph circulation. It is found that the ingestion of food invariably raises the arterial blood-pressure, and with it the capillary and venous blood-pressure, and thus by stimulating the heart, augmenting its output and contractile energy. With this rise in capillary pressure there is an exudation into the areolar spaces, and correspondingly a rise of the red corpuscles, hæmoglobin, and specific gravity in the blood. It is further found that various foods cause this rise of blood-pressure and lymph exudation in very various degrees, and in the following descending order—meat meal, ordinary meal, vegetable meal, and milk meal. On testing the different food elements it was discovered that cold water, starch, fats, gelatin, proteid as represented by myosin or egg albumen, sugars, pepsin, and hydrochloric acid, do not cause the blood-pressure or cause the flow of tissue-lymph. On the other hand, glycogen and lævulose decisively affects them. Sodium chloride raises, and potassium chloride depresses the arterial pressure. Uric acid, xanthin, creatinin, carnin, meat extracts raise the pressure and augment the lymph-flow, as also do tea, coffee, and cocoa. Thus bodies with a purin ($C_5H_4$) basis have a marked effect. It seems that food constituents themselves (proteids, fats, carbohydrates) do not possess the power of starting the mechanism by which lymph is dispensed to the tissues throughout the body, but that nature associates with our foodstuffs small quantities of very active substances which bring into play that mechanism, though these substances themselves are practically devoid of food value, and that man frequently increases this natural lymph stimulation by the use of salt and beverages containing bodies which also incite the flow of lymph. Exercise causes a marked rise in the arterial pressure, followed by a sudden fall and succeeded by a second slighter rise of pressure. The first rise is produced by the muscular contraction causing a partial or temporary occlusion of the intramuscular arteries and arterioles, and thus an increase in the peripheral resistance, and also by reflex cardiac stimulation. At the same time the capillary pressure in the contracting muscle falls and lymph will not be exuded, though elsewhere this occurs. When the contraction ceases the intramuscular capillary pressure rises and lymph is exuded into the muscle. Exercise thus greatly increases the lymph flow to the muscles ; it is removed partly by reabsorption into the blood-vessels and partially by flowing along the lymphatics. If exercise is prolonged the rise of arterial pressure gradually becomes less and less pronounced, and may in time be succeeded by a fall. This is due to the exuded lymph obstructing the pressure of the muscular fibres on the arterioles, and the tissues get lymph-logged. As is well known massage quickly disperses the lymph and thus restores the contractility of the muscles. During rest after exerci^e there is a steady and persistent rise of all t e blood-pressures and a corresponding effusion of tissue-lymph. The physiological intent of this is to repair and re-charge the muscular fibres. These changes are probably brought about by the influence of autogenous waste-products produced in contraction. It is interesting to observe that the characteristic double curve of arterial pressure produced by exercise and rest can also be produced by ammonium lactate and by creatin—substances which are believed to form stages in the passage of proteid towards urea in muscle metabolism. A large volume of lymph (not less than 20 per cent.) is exuded into the somatic and splanchnic tissues. The arterial pressure sinks very low, and the venous pressure rises to a high point—to a little over one-half of the arterial pressure, whereas in the waking state it is only one-fifth of that pressure. This effusion of lymph into the tissues is for the purpose of restoration of function. Rest differs from sleep, in that the vaso-motor mechanism is not relaxed, and lymph is then only exuded under the influence of lymph pressure agents, whether exogenous (as during digestion) or endogenous (after exercise). Carbonic acid raises the blood-pressure—even as much as 30 millimetres of mercury, as in a badly-ventilated room. On the other hand sulphurettted hydrogen lowers it. Tissue-lymph is a filtrate or pressure product; no evidence was forthcoming from the above experiments that the various substances which cause its flow from the capillaries to the tissue spaces do so, except by increasing the arterial pressure. In disease the state of the arterial system may be hypotonic or hypertonic—in the former, *e.g.*, neurasthenia, phthisis, etc., the effusions of tissue lymph are diminished; in the latter, *e.g.* gout, Bright's disease, they are in excess. Hence the value of diminishing the intake in gout of exogenous lymph factors. For instance, boiling meat removes the purin bodies, and correspondingly boiled meat increases the lymph flow to the tissues far less than does roast meat. This is far more important than confining the patient to white meats, which contain as much purins as do dark meats such as beef. Further, eggs, white bread, rice, tapioca, green vegetables, potatoes, butter and cheese, are free from purins, and so good foods for the gouty. Similarly salt should be prohibited, and its place taken by potassium chloride. Ammonium hippurate (in doses of 2 grains) reduces the arterial pressure, glycogen and lævulose raise it. The former is of service in gout, the latter in hypotonic conditions.

[2] Brit. Med. Jour., Sept. 17. [3] Med. Rec., May 14. [4] Lancet, April 30 and May 7.

*(To be continued.)*

---

